# Association of Insulin Resistance with Chronic Kidney Disease in Non-Diabetic Subjects with Normal Weight

**DOI:** 10.1371/journal.pone.0074058

**Published:** 2013-09-13

**Authors:** Shanying Chen, Youming Chen, Xinyu Liu, Mi Li, Bide Wu, Yongqiang Li, Yan Liang, Xiaofei Shao, Harry Holthöfer, Hequn Zou

**Affiliations:** 1 Department of Nephrology, Third Affiliated Hospital of Southern Medical University, Guangzhou, Guangdong, China; 2 Department of Nephrology, Zhangzhou Affiliated Hospital of Fujian Medical University, Zhangzhou, Fujian, China; 3 Clinical Laboratory, Third Affiliated Hospital of Southern Medical University, Guangzhou, Guangdong, China; 4 Blood purification center, No. 5 Affiliated Hospital of Sun Yat-sen University, Zhuhai, Guangdong, China; 5 National Centre for Sensor Research/BioAnalytical Sciences, Dublin City University, Dublin, Ireland; University of Warwick – Medical School, United Kingdom

## Abstract

**Objective:**

To the best of our knowledge, the association of insulin resistance (IR) with chronic kidney disease (CKD) has not been well studied in normal-weight individuals. The aim of this study is to examine whether IR is associated with CKD in non-diabetic subjects with normal weight. We also examine whether the presence of obesity modifies the association of IR with CKD.

**Methods:**

Data were drawn from a cross-sectional survey in China. Both estimated glomerular filtration rate and urinary albumin to creatinine ratio were used as markers of CKD. Logistic regression models and the quartiles of homeostatic model assessment of insulin resistance were used to explore the associations of IR with CKD in entire cohort, normal-weight and overweight/obese subpopulations.

**Results:**

In normal-weight subpopulation, the prevalence of IR and metabolic syndrome were 11.11% and 8.99%, respectively. In the entire cohort, the highest quartile HOMA-insulin resistance had a 70% increased risk for CKD (RR 1.70, 95% CI 1.07, 2.71, P=0.03, comparing the highest to the lowest quartile). However, when adding obesity to the model, the association was abolished. IR was associated with CKD in overweight/obese subpopulation but not in normal-weight subpopulation.

**Conclusion:**

IR and MetS in normal-weight individuals is common in the Chinese population. IR is associated with CKD in overweight/obese subpopulation but not in normal-weight subpopulation and the presence of obesity modifies the association of IR with CKD.

## Introduction

Insulin resistance (IR) is a hallmark of metabolic syndrome and mostly caused by abdominal obesity [1]. But IR can also occur in normal-weight individuals, the existence of a subgroup of individuals with normal weight but with metabolic disturbances usually associated with obesity was first suggested in 1981 by Ruderman et al [2,3]. Such metabolically obese, normal weight (MONW) individuals are common in the general population. Like people with overt obesity, MONW individuals are hyperinsulinemic, insulin-resistant, and predispose to type 2 diabetes, hypertriglyceridemia, and premature coronary heart disease. They probably represent one end of the spectrum of people with the insulin resistance syndrome [4,5].

IR is associated with kidney damage in obese individuals and patients with diabetes [1,6,7,8]. To the best of our knowledge, the association of IR with chronic kidney disease (CKD) has not been well studied in normal-weight individuals. Whether MONW individuals predispose to CKD is unclear. The aim of this study is to examine whether insulin resistance is associated with CKD in non-diabetic subjects with normal weight. We also examine whether the presence of obesity modifies the association of IR with CKD.

## Methods

### Subjects

This study was approved by the ethics committee of the Third Affiliated Hospital of Southern Medical University. All subjects gave their written informed consent. Data were drawn from a population-based, cross-sectional survey conducted in Wanzhai Town, Zhuhai City. Zhuhai is located on the southern coast of China. There are six communities in Wanzhai Town and three of them were randomly selected for this survey. This survey was conducted between June, 2012, and October, 2012. All adult residents (aged 18 years or older) living in the three communities were invited to participate in this survey. Participants were recruited by mail and home visits. 2142 residents voluntarily participated in the survey. We excluded subjects with diabetes.

### Data collection

All doctors, medical students and nurses participating in the study had received intensive training. Data were collected in a local community clinic, health stations or during home interview. Data on age, sex, education, current or past cigarette smoking, alcohol use, physical activity, dietary habits, personal history, family history and medication history were obtained through questionnaires.

### Physical examination

Blood pressure was determined with a calibrated mercury sphygmomanometer in a seated position after at least 5 minutes rest. Blood pressures were determined three times and the average of the three reading was calculated. Anthropometric indices were collected in the community clinics and measured according to the recommendation by the World Health Organization. Waist circumference was measured at the level of midway between the lower rib margin and the iliac crest in the midaxillary line, with the participants standing with light garments and breathing out gently. Hip circumference was measured at the widest point around the buttocks [9]. BMI was calculated as weight (in kilograms) divided by the square of the height (in meters).

### Laboratory variables

All blood specimens were collected after an overnight fast for at least 10 hours in a local community clinic. First morning urine samples were collected. Women who were actively menstruating were excluded from the urine test. We also excluded individuals having urinary tract infection symptoms. All specimens from collection sites were transported to the central laboratory in the Third Affiliated Hospital of Southern Medical University in 3 hours and stored at 2-8°C until analysis.

Urinary albumin was measured by an immune nephelometric method. Serum insulin was measured using electrochemiluminescence immunoassays. Urinary creatitine, serum creatinine, fasting glucose, serum total cholesterol, serum triglyceride and serum high density lipoprotein cholesterol were measured by colorimetric methods. High sensitivity C-reactive protein (CRP) was measured using an enzymatic immunoassay turbidimetric method.

Homeostatic model assessment of insulin resistance (HOMA-IR) was calculated as fasting plasma glucose (mmlo/L) ×fasting insulin (mU/L)/22.5 [10]. Glomerular filtration rate was estimated using the four-variable Modification of Diet in Renal Disease (MDRD) equation [175× (Scr)-1.234 × (Age)-0.179 ×(if female, ×0.79)] [11]. Urinary albumin to creatinine ratio (ACR, mg/g) was calculated as the ratio of urinary albumin to urinary creatitine.

### Definitions

Normal weight was defined as BMI < 24 kg/m^2^, and obesity was defined as BMI ≥ 28 kg/m^2^. Overweight was defined as BMI ≥ 24 kg/m^2^ and BMI < 28 kg/m^2^ [12]. Insulin resistance was defined as exceeding the 75% percentile of HOMA-IR in normal glucose tolerance subjects. According to an epidemiological survey in China, the cut-off point is 2.69 [13]. CKD was defined as an eGFR less than 60 ml/min/1.73m2 and/or ACR ≥30mg/g [14]. Diabetes mellitus was defined as a fasting serum glucose ≥7.0 mmol/l and/or self-reported diagnosis of diabetes. Metabolic syndrome (MetS) was defined as having at least three of the following five criteria: abdominal obesity, elevated triglyceride levels (≥ 150 mg/dl), low high density lipoprotein cholesterol levels (<40mg/dl in men, or <50mg/dl in women), an elevated blood pressure (≥130 ⁄ 85 mmHg) and an elevated fasting glucose level (≥ 110 mg⁄dl or 6.1 mmol⁄ l) [15].

### Data analysis

#### Using *Stata* (*version 11*) for data analysis.

Continuous variables were shown as mean ± standard deviation if they had a normal distribution. Median and interquartile range were used to show continuous variables having a skewed distribution. The categorical variables were presented as absolute and relative (%) values. A two-tailed p value <0.05 was considered significant.

Baseline characteristics of subjects were examined in both normal-weight and overweight/obese subpopulations. Based on HOMA index, both normal-weight and overweight/obese subjects were divided into IR and insulin sensitive subgroups. Baseline characteristics of two subgroups were also examined. Student’s t test or Wilcoxon rank-sum test was used for continuous variables and the chi-squared test or Fisher’s exact test was used for categorical variables. Prevalence of IR, CKD and MetS were also calculated and tested using chi-squared test.

In order to examine whether IR is associated CKD in entire cohort, normal-weight and overweight/obese subpopulations, logistic regression models were used. HOMA-IR was divided into quartiles and used as a independent variable. The first model was adjusted for age and sex. In model 2, cormobidities (history of hypertension, history of coronary heart disease, history of stroke), lifestyle factors (current smoking, current alcohol use, physical inactivity), education attainment and taking Chinese medicine were included. To examine the effect modification by obesity on associations of IR with CKD, obesity was included in the next model. Components of MetS, including an elevated triglyceride level, a low high density lipoprotein cholesterol level, an elevated blood pressure, and an elevated fasting glucose level are potentially down stream consequences of IR [1] and also potentially in the causal pathway between IR and CKD. So they were not included in the adjusted models.

Logistic regression analyses were also conducted separately in normal-weight and overweight/obese subpopulations.

## Results

There were 2142 study subjects (the mean age was 49.55 ± 13.44 years and 796 were men) and 308 subjects were excluded because of missing data for serum insulin, serum fasting glucose, serum creatitine, CRP, urinary albumin, urinary creatitine, BMI, or waist circumference. We also excluded 151 subjects with diabetes. Among 1683 non-diabetic subjects, the mean age was 51.98 ± 14.54 years and 36.67% (617) were men. The employment rate was higher in men and employers offered free physical examinations. It led to a lower participation rate for men. 648 subjects had a higher BMI (≥ 24 kg/m^2^), and the prevalence of overweight/obesity was 38.50%. 128 subjects were taking antihypertensive agents, 3 subjects were taking antiretroviral drugs for hepatitis B, 2 subjects were taking sleeping pills, and 23 subjects were taking Chinese Medicine Patent Prescription. No subjects used contrast agents in the past three months or used antibiotics. No subjects had history of recurrent urinary tract infections and obstructive uropathy in this population

### Baseline characteristics of normal-weight and overweight/obese subpopulations (Table 1)

Characteristics of normal-weight and overweight/obese subpopulations in the entire cohort are presented in table 1. Overweight/obese subpopulation had significantly higher BMI and larger waist circumference than normal-weight subpopulation (P< 0.001). In general, overweight/obesity was associated with high blood pressure, high blood levels of fasting glucose, uric acid, triglyceride, high levels of ACR, and high blood levels of CRP.

**Table 1:** Baseline characteristics^**a**^ of subjects according to body mass index

	**Normal-weight**	**Overweight/Obese**		
	**n = 1035**	**n = 648**		**P Value**
HOMA – IR (uU/ml .mmol/ml)	1.42 (1.00 - 1.99)	2.41 (1.70 - 3.52)		<0.001
Insulin Resistance (%)	115 (11.11)	274 (42.28)		<0.001
**Demographics**				
Age (Years)	50.83 ± 15.54	53.83 ± 12.58		<0.001
Male (%)	375 (36.23)	272 (26.28)		<0.001
**Clinical Characteristics**				
Body Mass Index (kg/m2)	21.08 ± 1.98	26.57 ± 2.30		<0.001
Waist circumference(cm)	77.50 ± 7.62	90.52 ± 7.78		<0.001
History of Hypertension (%)	123 (11.88)	179 (27.62)		<0.001
History of coronary heart disease (%)	17 (1.64)	15 (2.31)		0.36
History of stroke (%)	2 (0.19)	3 (0.44)		0.38
Current smoker (%)	122 (11.79)	87 (13.43)		0.31
Current alcohol use (%)	53 (5.12)	41 (6.32)		0.29
Education attainment High school or above(%)	467 (45.12)	245 (37.81)		<0.001
Physical inactivity (%)	585 (56.52)	370 (57.10)		0.82
Systolic blood pressure (mm Hg)	124.01 ± 19.55	133.51 ± 19.25		<0.001
Diastolic blood pressure (mmHg)	75.47 ± 10.76	81.51 ± 10.34		<0.001
**Laboratory**				
Serum creatitine (umol/L)	71.40 ± 16.01	75.30 ± 16.39		<0.001
eGFR ( ml/min/1.73m^2^)	102.57 ± 22.59	96.41 ± 20.59		<0.001
Serum uric acid (umol/L)	331.01 ± 88.45	378.48 ± 98.18		<0.001
ACR (mg/g))	7.96 (5.48 - 12.38)	8.97（5.92 - 15.82）		<0.001
Fasting glucose (mmo/l)	4.68 ± 0.47	4.92 ± 0.61		<0.001
Serum C-reactive protein (mg/l)	0.67 (0.33 - 1.53)	1.63（0.82 - 3.34）		<0.001
Serum triglyceride (mmol/L)	1.05 (0.77 - 1.46)	1.55 (1.08 - 2.19)		<0.001
Serum low density lipoprotein (mmol/l)	3.08 ± 0.89	3.31 ± 0.90		<0.001
Serum high density lipoprotein (mmol/l)	1.60 ± 0.33	1.45 ± 0.28		<0.001

^a^ Mean ± SD or median (25th to 75th percentiles) for continuous variables and absolute and relative (%) values for category variables are presented.

HOMA-IR: Homeostatic model assessment of insulin resistance; eGFR: estimated Glomerular filtration rate

ACR: Urinary albumin to creatinine ratio

### Baseline characteristics of insulin sensitive and IR subjects according to BMI (Table 2)

Characteristics of insulin sensitive and insulin resistance subjects according to BMI were presented in table 2. In both subpopulations, IR subgroup had a higher BMI, a larger waist circumference, a higher diastolic blood pressure, high blood levels of fasting glucose, triglyceride, higher levels of ACR, and high blood levels of CRP than insulin sensitive subgroup (P< 0.001).

**Table 2:** Baseline characteristics^**a**^ of insulin sensitive and insulin resistance subjects according to body mass index

	**Normal-weight**			**Overweight/Obese**			
	**Insulin sensitive**	**Insulin resistance**	**P value**	**Insulin sensitive**		**Insulin resistance**	**P value**
	**n = 920**	**n = 115**		**n = 374**		**n = 274**	
HOMA – IR (uU/ml .mmol/ml)	1.33 (0.95 - 1.75)	3.25 (2.87 - 4.03)	<0.001	1.81 (1.32 - 2.17)		3.78 (3.11 - 4.66)	<0.001
**Demographics**							
Age (Years)	50.36 ± 15.61	54.55 ± 14.49	0.006	53.45 ± 12.25		54.34 ± 13.02	0.38
Male (%)	316 (34.34)	29 (25.22)	0.05	154 (41.18)		118 (43.07)	0.63
**Clinical Characteristics**							
Body Mass Index (kg/m2)	20.97 ± 1.98	21.96 ± 1.70	<0.001	26.07 ± 1.93		27.25 ± 2.57	<0.001
Waist circumference(cm)	77.00 ± 7.54	81.42 ± 7.09	<0.001	88.96 ± 7.18		92.66 ± 8.07	<0.001
History of Hypertension (%)	100 (10.87)	23 (20)	0.04	86 (22.99)		93 (33.94)	0.002
History of coronary heart disease (%)	15 (1.63)	2 (1.74)	1	6 (1.60)		9 (3.28)	0.19
History of stroke (%)	2 (0.22)	0	1	1 (0.27)		2 (0.73)	0.58
Current smoker (%)	114 (12.39)	8 (7.00)	0.09	50 (13.37)		37 (13.50)	0.98
Current alcohol use (%)	52 (5.65)	1 (0.87)	0.03	31 (8.29)		10 (3.65)	0.02
Education attainment High school or above (%)	420 (45.65)	47 (40.87)	0.33	146 (39.04)		99 (36.13)	0.45
Physical inactivity (%)	525 (57.07)	60 (52.17)	0.32	207 (55.35)		163 (59.49)	0.29
Systolic blood pressure (mm Hg)	123.25 ± 19.53	129.88 ± 18.79	< 0.001	131.44 ± 19.44		136.36 ± 18.67	0.002
Diastolic blood pressure (mmHg)	75.06 ± 10.77	78.59 ± 10.24	0.001	80.13 ± 10.62		83.40 ± 6.64	<0.001
**Laboratory**							
Serum creatitine (umol/L)	71.51 ± 16.23	70.56 ± 14.15	0.55	73.93 ± 16.06		77.16 ± 16.69	0.01
eGFR( ml/min/1.73m2)	102.86 ± 22.41	100.25 ± 23.86	0.24	98.34 ± 20.23		93.78 ± 20.82	0.005
Serum uric acid (umol/L)	328.34 ± 87.24	352.32 ± 95.35	0.006	358.52 ± 92.95		405.73 ± 98.73	<0.001
ACR (mg/g)	7.78 (5.48 - 12.02)	9.99 (6.36 - 15.65)	0.001	8.49 (5.57 - 13.61)		9.99 (6.36 - 19.45)	0.002
Fasting glucose (mmo/l)	4.63 ± 0.43	5.10 ± 0.57	<0.001	4.71 ± 0.48		5.21 ± 0.64	<0.001
Serum C-reactive protein (mg/l)	0.63 (0.32 - 1.45)	0.96（0.53 - 2.67）	<0.001	1.35（0.71 - 2.75）		2.20 (1.04 - 3.86)	<0.001
Serum triglyceride (mmol/L)	1.02 (0.76 - 1.37)	1.46 (1.10 - 2.45)	<0.001	1.46 (1.10 - 2.45)		1.36 (0.97 - 1.90)	<0.001
LDL (mmol/l)	3.08 ± 0.88	3.14 ± 0.95	0.95	3.34± 0.85		3.26 ± 0.97	0.27
HDL (mmol/l)	1.61 ± 0.33	1.49 ± 0.32	0.002	1.50 ± 0.30		1.37 ± 0.23	<0.001

^a^ Mean ± SD or median (25th to 75th percentiles) for continuous variables and absolute and relative (%) values for category variables are presented.

HOMA-IR: Homeostatic model assessment of insulin resistance; eGFR: estimated Glomerular filtration rate

ACR: Urinary albumin to creatinine ratio; LDL:Serum low density lipoprotein;HDL:Serum high density lipoprotein

### Prevalence of IR, MetS and CKD

**Figure 1:**
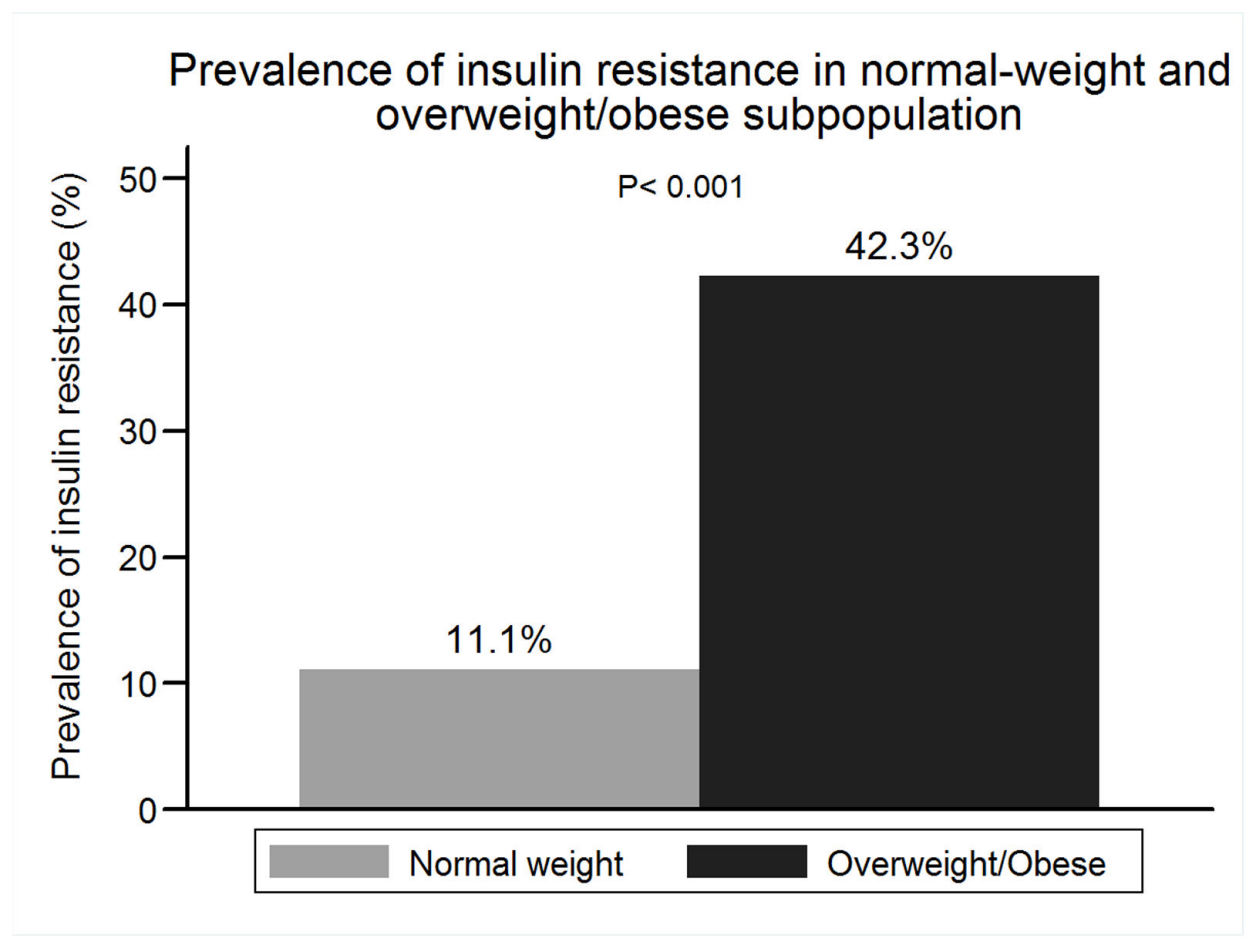
Prevalence of insulin resistance in normal-weight and overweight/obese subpopulation.

**Figure 2:**
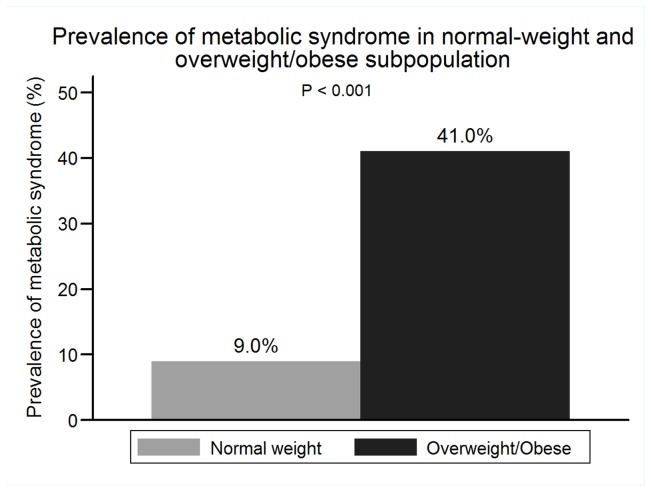
Prevalence of metabolic syndrome in normal-weight and overweight/obese subpopulation.

The prevalence of IR in the entire cohort, normal-weight subpopulation and overweight/obese subpopulation were 23.11% (389), 11.11% (115) and 42.28% (274), respectively (Figure 1). The prevalence of MetS was lower than that of IR, and 21.33% (359) subjects had MetS in the entire cohort. 8.99% normal-weight subjects had MetS. Overweight/obese subpopulation had a higher prevalence of IR and MetS than normal-weight subpopulation (Figure 2). 30.43% normal-weight subjects with IR had MetS and 61.68% overweight/obesity subjects with IR had MetS

**Figure 3:**
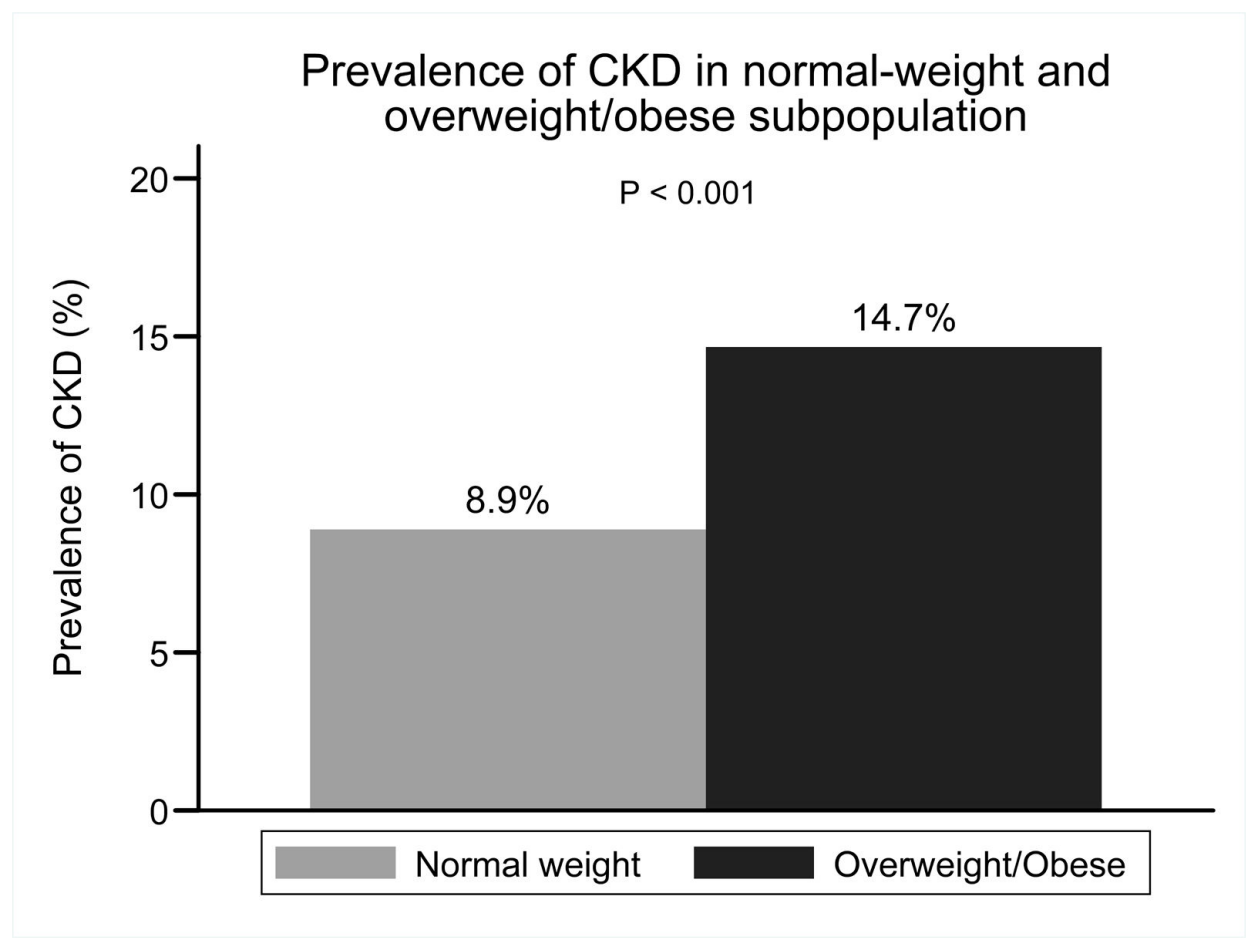
Prevalence of CKD in normal-weight and overweight/obese subpopulation.

**Figure 4:**
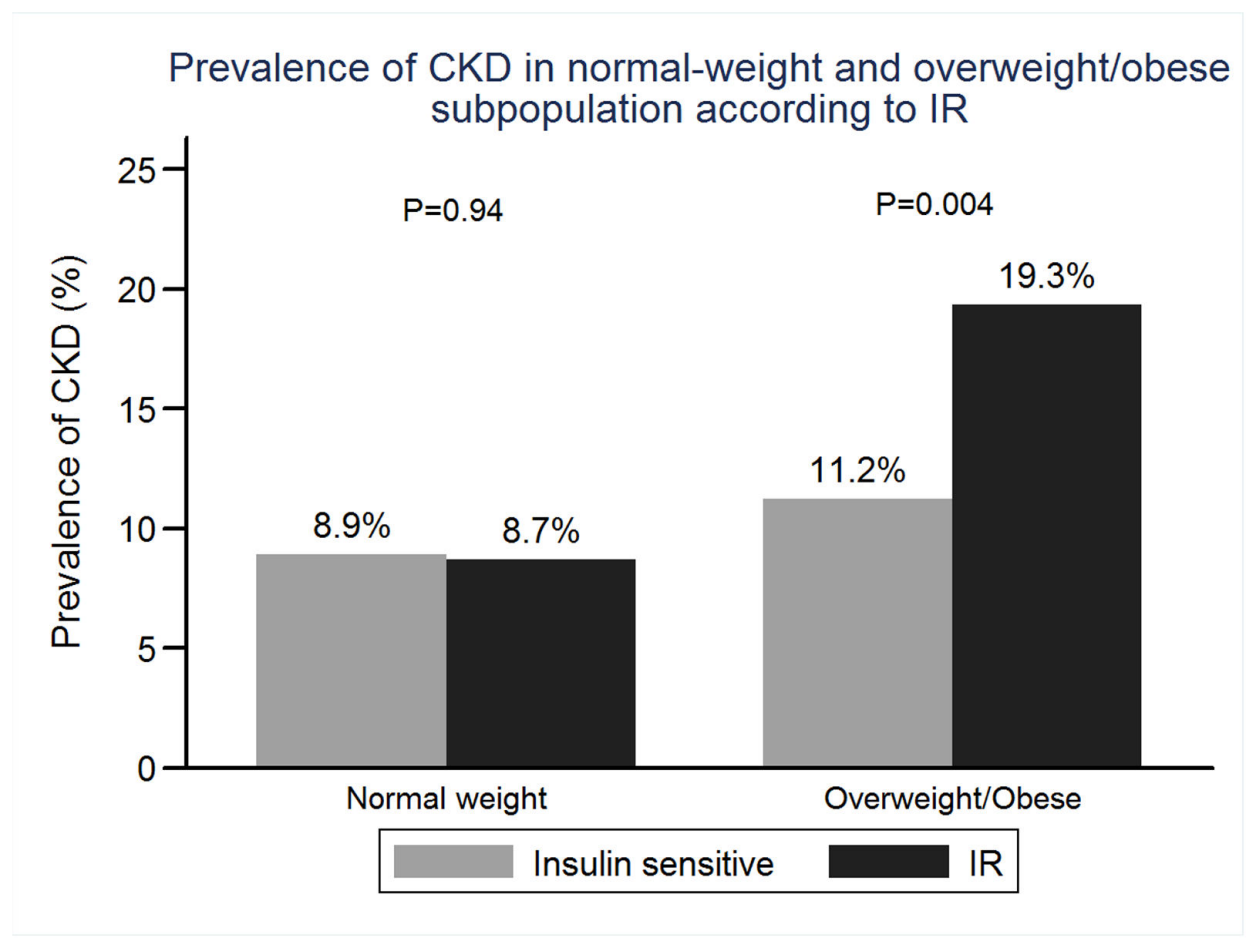
Prevalence of CKD in normal-weight and overweight/obese subpopulation according to IR.

In the entire cohort, 187 subjects had CKD. Only 34 (2.02%) subjects had reduced eGFR (defined as eGFR <60 ml/min/1.73m2). No subjects needed dialysis treatment. The prevalence of CKD in the entire cohort, normal-weight subpopulation and overweight/obese were 11.11% (187), 8.89% (92) and 14.66% (95), respectively (Figure 3). There was no significant difference in prevalence of CKD between IR and insulin sensitive subgroups in normal-weight subpopulation (8.91% vs 8.70%, P= 0.94). But in overweight/obese subpopulation, IR subgroup had significantly a higher prevalence of CKD than insulin sensitive subgroup (11.23% vs 19.34%,P=0.004.) (shown in the Figure 4).

### Associations of IR with CKD in entire cohort, normal-weight and overweight/obese subpopulations

After adjustment for age and sex, IR was significant associated with CKD (RR 2.07, 95% CI 1.32, 3.26, P=0.002, comparing the highest to the lowest quartile) (table 3). After further adjustment for history of hypertension, history of coronary heart disease, history of stroke, current smoking, current alcohol use, physical inactivity, education attainment and taking Chinese medicine, the association of IR with CKD was also significant. The highest quartile HOMA-insulin resistance had a 70% increased risk for CKD (RR 1.70, 95% CI 1.07, 2.71, P=0.03). However, when adding obesity to the model, the association was abolished (RR 1.43, 95% CI 0.87, 2.36, P=0.16, comparing the highest to the lowest quartile). After adjustment for the potential confounders, age and history of hypertension were significantly associated with CKD in entire cohort.

**Table 3:** Association of insulin resistance with CKD in entire cohort.

	**Model one ^a^**		**Model two ^b^**		**Model three ^c^**	
	**RR (95% CI**)	**P value**	**RR (95% CI**)	**P value**	**RR (95% CI**)	**P value**
**Quartile one(n=421**)	**Reference**		**Reference**		**Reference**	
HOMA-IR < 1.182						
**Quartile two (n=421**)	1.17	0.56	1.11	0.7	1.08	0.76
HOMA-IR:1.182-1.711	(0.70, 1.94)		(0.66, 1.85)		(0.65, 1.81)	
**Quartile three (n=421**)	1.63	0.04	1.50	0.10	1.33	0.26
HOMA-IR:1.711-2.597	(1.12, 2.63)		(0.92, 2.43)		(0.81, 2.20)	
**Quartile four (n=420**)	2.07	0.002	1.70	0.03	1.43	0.16
HOMA-IR > 2.597	(1.32, 3.26)		(1.07, 2.71)		(0.87, 2.36)	

**a** Adjusted for age, sex

b Adjusted for age, sex, history of hypertension, history of coronary heart disease, history of stroke, current smoker, current alcohol use, physical inactivity, education attainment, and taking Chinese medicine

c Adjusted for above + obesity

As shown in table 4, the association of IR with CKD in normal-weight subpopulation was not significant. However in overweight/obese subpopulation, in a similar adjusted model, IR was associated with CKD (in the highest quartile HOMA-insulin resistance, RR 2.10, 95% CI 1.11, 3.97, P=0.02). Age, history of hypertension and history of coronary heart disease were associated with CKD in overweight/obese subpopulation.

**Table 4:** Association of insulin resistance with CKD in normal-weight and overweight/obese subjects.

	**Normal weight**				**Overweight/obese**			
	**Model one** ^a^		**Model two** ^b^		**Model one** ^a^		**Model two** ^b^	
	RR (95% CI)	P value	RR (95% CI)	P value	RR (95% CI)	P value	RR (95% CI)	P value
Quartile one	Reference		Reference		Reference		Reference	
Quartile two	0.75	0.39	0.71	0.33	0.66	0.26	0.75	0.46
	(0.38, 1.45)		(0.36, 1.41)		(0.32, 1.37)		(0.35, 1.59)	
Quartile three	0.86	0.66	0.88	0.71	1.14	0.69	1.09	0.81
	(0.45, 1.67)		(0.45, 1.72)		(0.59, 2.20)		(0.55, 2.12)	
Quartile four	1.31	0.37	1.19	0.57	1.98	0.03	2.10	0.02
	(0.73, 2.37)		(0.64, 2.22)		(1.07, 3.64)		(1.11, 3.97)	

**a** Adjusted for age, sex

b Adjusted for age, sex, history of hypertension, history of coronary heart disease, history of stroke, current smoker, current alcohol use, physical inactivity, education attainment, and taking Chinese medicine

## Discussion

The results of the current study suggest that IR was associated with CKD in overweight/obese subpopulation but not in normal-weight subpopulation and the presence of obesity modifies the association of IR with CKD.

A number of interrelated factors may contribute to the pathogenesis of hyperinsulinemia and insulin resistance. They include obesity, low birth weight, physical inactivity, and family history [1,4]. In the review of Bagby, the detailed pathogenesis of IR initiated by obesity was described. Increased fat mass yields high circulating free fat acids, fat acid and its metabolites lead to serine/threonine phosphorylation of insulin receptor substrate-1 on serine. As a result, insulin receptor activation is reduced [1].

However, Ruderman first proposed metabolically obese in normal weight (MONW) individuals. Based on a study for National Health and Nutrition Examination Survey III, 4.6% normal-weight men and 6.2% normal-weight women met the diagnostic criteria of MetS [3,5]. Our study also suggests that MONW are common in the Chinese population. 11.11% normal-weight subjects had IR and 8.99% had MetS. Although they had normal weight, they presented highinsulinemia and/or IR as well as hypertriglyceridemia and hypertension [2,3].

Although IR can occur in normal weight individuals, the pathogenesis is unknown [7]. A plausible explanation is that greater whole stored body fat is associated with IR despite of normal BMI range [4,5,7]. According to one prior study, even there were no significant differences in birth weight and BMI between IR and insulin sensitive subgroups, IR subgroup had higher fat mass measured by dual energy X-ray absorptiometry [16]. In another study in non-obese, normoglycemic subjects, both IR and insulin sensitive subgroups had normal BMI and there was no difference in BMI between two subgroups. Body adipose stores were significantly increased in the IR subgroup compared to the insulin sensitive subgroup [17]. Whether the slight expansion of abdominal adipose can lead to metabolic disorders needs to be further explored. Apart from fat mass and fat distribution, dietary, physical inactivity, genetic factors are associated with IR in non-obese individuals [1,18].

In the current study, the IR subgroup had a higher BMI and a larger waist circumference than the insulin sensitive subgroup in both normal-weight and overweight/obese subpopulations. The results suggest that fat mass is associated with insulin resistance even in normal-weight subjects. But we did not find that the slight expansion of fat mass contributes to CKD in normal-weight subgroup.

Evidence suggests that insulin resistance precedes and probably contributes to the development of microalbuminuria in type 1 diabetic patients [19], type 2 diabetic patients [20] and hypertensive patients [21]. Data from epidemiological studies suggest that insulin resistance and the cluster of abnormalities related to the metabolic syndrome are associated with microalbuminuria and reduced glomerular filtration rate [6,22,23,24,25]. A large prospective longitudinal study using data from the Atherosclerosis Risk in Communities (ARICs) cohort indicated that MetS and HOMA insulin resistance quintiles were associated with the incidence of CKD in nondiabetic adults, ever after controlling for the development of diabetes mellitus and hypertension [26]. The similar results were obtained in the current study, and the highest quartile HOMA-insulin resistance had a 70% increased risk for CKD. And IR promotes kidney disease by worsening renal hemodynamics, releasing inflammatory cytokine and renal endoplasmic reticulum stress. Kidney damage induced by IR includes glomerular hyperfiltration, endothelial dysfunction, increased vascular permeability, increased glomerular capillary pressure, protein traffic, mesangial hyperplasia, renal hypertrophy, increased endothelial cell proliferation and so on [27,28].

IR is the core of MetS, other components of MetS are potentially down stream consequences of IR [1]. The constellation of abnormalities related to insulin resistance including those clustering in the metabolic syndrome, adipocytokine dysregulation, hyperinsulinemia and low-grade inflammation are all involved in worsening kidney function [26-28]. Other components of MS and the number of MS are associated with the incidence of CKD. Each single component of MetS may in fact play a role in kidney damage [26].

The results of the current study also suggest that obesity modifies the association of IR with CKD. After adjustment for obesity, the association of IR with CKD was not significant. These data suggest a strong role of obesity in developing of CKD in subjects with IR. Whether genetic factors, physical inactivity and dietary can have a role in the interaction with the association of IR with CKD needs to be further explored. The results are different from a previous study. In the Atherosclerosis Risk in Communities (ARICs) study, IR is associated with the incidence of CKD and this association is independent of BMI. In this previous study, all subjects were black or white and aged 45-64 years [26]. The differences in race and age should be considered. Another study of Chinese subjects also showed that IR was associated with the prevalence of CKD and rapid decline in renal function. But this study only concerned elderly individuals [29]

Previous longitudinal studies showed that MONW individuals predispose to type 2 diabetes and premature coronary heart disease [30]. However, to our knowledge, whether normal-weight individuals with IR predispose to kidney damage is unclear. The results of the current study suggest that IR is not significantly asscociated with CKD in normal-weight subpopulation. Metabolic syndrome and its components are associated with chronic kidney disease (CKD) development. Previous studies has shown that abdominal obesity, elevated triglyceride levels, low high density lipoprotein cholesterol levels, an elevated blood pressure and an elevated fasting glucose level were the components that increased the risk of CKD [26]. Among normal-weight subjects with IR, 30.43% had MetS. In the same population, 61.68% overweight/obese subjects with IR had MetS. A relatively low prevalence of MetS might contribute to negative results.

The first limitation of this study is its cross-sectional survey and lacking of longitudinal data, so we cannot infer causality. IR is associated with an increased risk for CKD in nondiabetic patients and is also common in patients with mild-to-moderate stage CKD [27]. We need longitudinal data to demonstrate the association of IR and CKD. Second, the gold standard for investigating and quantifying insulin resistance is hyperinsulinemic euglycemic clamp. However, homeostatic model assessment of insulin is a reliable indicator of insulin resistance [9]. Third, all the indicators of CKD (eGFR and ACR) were obtained on the basis of single measurements without repeating tests. According to the Nhanes III data in U.S.A, only 63.2% individuals with microalbuminuria still had albuminuria in the second visit [31]. Finally, only 36.67% of the subjects were men, the sample is biased.

## Conclusion

IR and MetS in normal-weight individuals are common in the Chinese population. IR is associated with CKD in overweight/obese subpopulation but not in normal-weight subpopulation and the presence of obesity modifies the association of IR with CKD.
